# Iatrogenic IVC Perforation after Successful Catheter-Directed Thrombolysis 

**DOI:** 10.1155/2017/3746815

**Published:** 2017-08-29

**Authors:** Ata Firoozi, Jamal Moosavi, Omid Shafe, Parham Sadeghipour

**Affiliations:** ^1^Rajaie Cardiovascular Medical and Research Center, Iran University of Medical Sciences, Tehran, Iran; ^2^Cardiovascular Intervention Research Center, Rajaie Cardiovascular Medical and Research Center, Iran University of Medical Sciences, Tehran, Iran

## Abstract

Central vein perforation as a rare complication of venous interventions is considered a nightmare if occurring in thoracic cage but behaves benignly in abdominal or pelvic region. This is not a rule, as we unfortunately encountered during the procedure of venous intervention in our patient. Although mechanical control of iatrogenic perforation or rupture is the first and most critical step during interventional procedures, the importance of anticoagulant and thrombolytic agents reversal should not be overlooked.

## 1. Introduction

Venous intervention gradually becomes a major field of cardiovascular interventionists. There are generally four accepted goals for the treatment of lower extremity deep vein thrombosis (LEDVT): (1) diminish the severity and duration of symptoms; (2) prevent pulmonary embolism; (3) minimize the risk of recurrent venous thrombosis; and (4) prevent the postthrombotic syndrome (PTS).

As relief of outflow obstruction is one of the primary goals of therapy for LVEDT, this unfortunately is not accomplished by anticoagulation alone, as thrombus regression occurs in only 50% of patients [1]. According to recent studies, in acute phase of DVT, extensive and centrally located thrombus showed the highest probability in developing severe PTS and disease specific lower quality of life [2].

## 2. Case Presentation

A 47-year-old hairdresser lady referred to our center with documented diagnosis of extensive vein thrombosis involving the left iliofemoral system according to duplex ultrasound. The patient symptoms including abrupt-onset swelling and crushing pain of left leg began one week ago. Her detailed history lacked any medical admission or consumption of special drugs including hormonal contraceptives. For more delineation of thrombosis propagation we decided to evaluate her precisely using MR venography with gadolinium which revealed “filling defects in the left common and external iliac, common, and superficial femoral and popliteal veins in favor of acute extensive DVT.”

Laboratory data was not striking except the level of serum D-dimer (5297 mg/dl). Ongoing and annoying course of patient symptoms and massive burden of thrombosis, in addition to fear of future postthrombotic complications which possibly interfere with our patient occupation, altogether forced the treatment team to choose a definite therapy for her lower extremity DVT.

**Figure 1:**
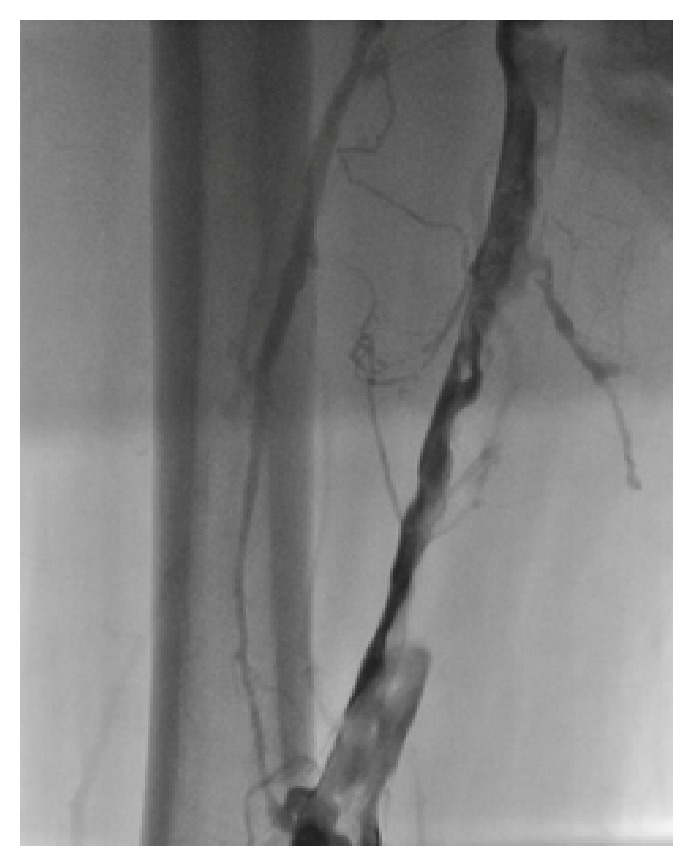
Thrombotic occlusion of femoral vein (patient in prone position).

The access site to the patient left iliofemoral DVT chosen to be ipsilateral popliteal vein with the patient prone on the angiographic table under ultrasound guidance. After successful cannulating the popliteal vein, contrast injection showed totally thrombotic occlusion of left common femoral and iliac veins but obviously patent IVC (Figure 1). The next step was installation of ultrasound-based infusion system, Lysus Infusion System (EKOS corporation, Bothell, WA) which delivered alteplase via a catheter with one central lumen and three separate infusion ports (total dose 1 mg/hour). It combines high-frequency lower-power ultrasound with simultaneous catheter-directed thrombolytics to accelerate clot dissolution [3]. Also heparin was infused (250 U/hour) in conjunction.

**Figure 2:**
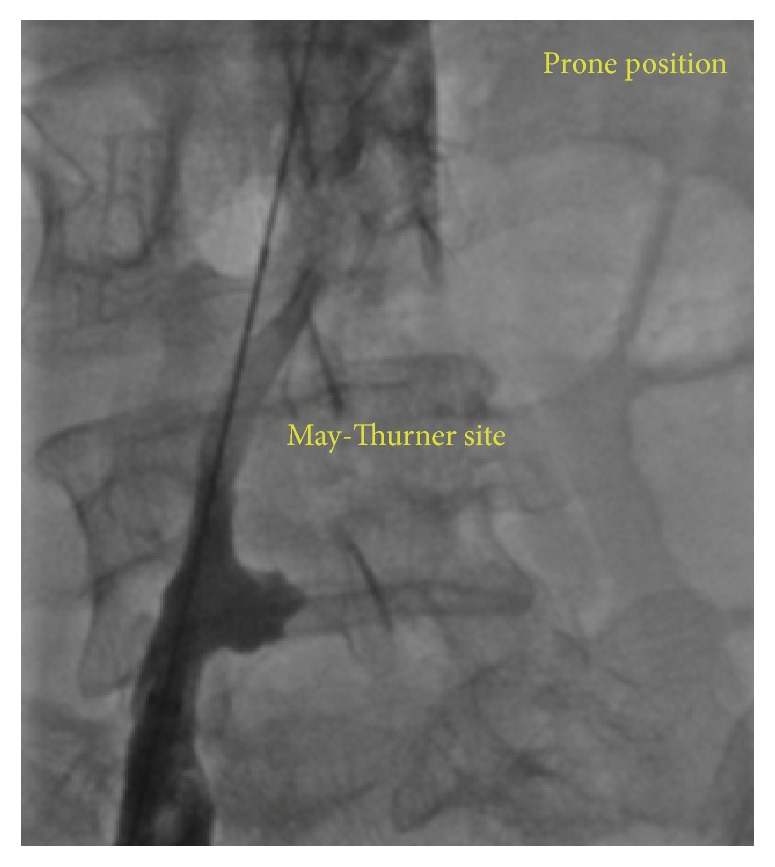
Follow-up venography revealed dramatic resolution of clot burden.

In the following day, the patient was brought back to angiography laboratory to perform follow-up venography which revealed dramatic resolution of clot burden and nice penetration of contrast toward patent IVC (Figure 2). Also, this diagnostic view demonstrated etiologic background of patient nonevoked extensive DVT which was significant stenosis of common iliac vein (May-Thurner syndrome).

**Figure 3:**
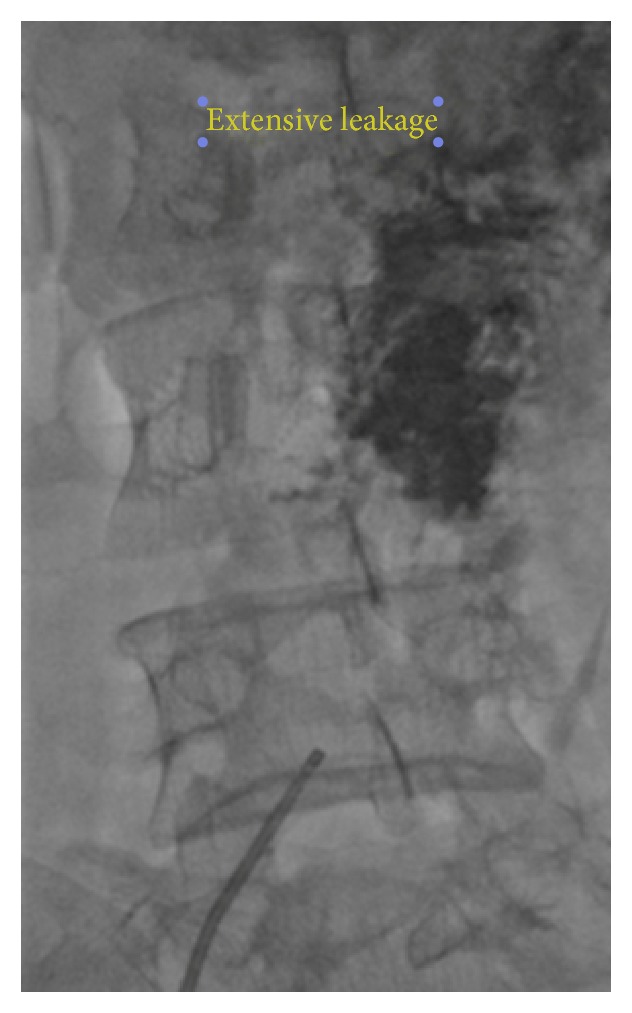
Leakage out of caveofemoral junction extensively.

During catheter passage and wire exchange, before deploying of stent, unfortunately we encountered leakage out of caveofemoral junction (the distal edge of IVC) extensively (Figure 3). At this critical moment, we decide to complete the stenosis stenting process, with this rational explanation that bleeding in venous system is benign and generally self-limiting. Thereafter, the patient returned to formal access supine position; the right venous femoral access was obtained quickly. We sent via this new venous route and previous popliteal vein access a BIB of size 24 and inflated it frequently in intervals of 5 to 25 minutes with hope of bleeding site sealing.

**Figure 4:**
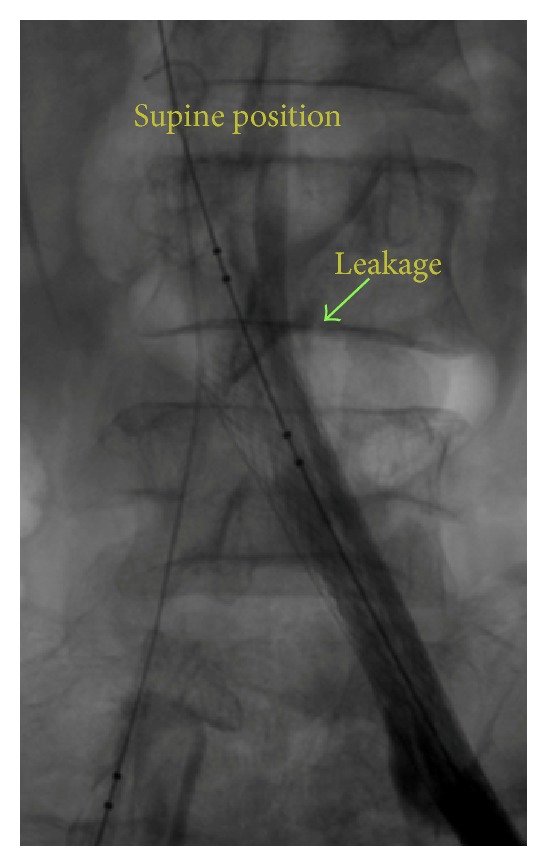
Unsuccessful result of balloon inflations forced us to decide to reverse anticoagulant condition of our patient (supine position).

In spite of patient stable hemodynamics, hemoglobin level dropped dramatically (6.5 g/dL from 11 g/dL at the beginning). Unsuccessful result of balloon inflations which was showed in subsequent injections (Figure 4) forced us to decide to reverse anticoagulant condition of our patient (due to alteplase and heparin infusion). We did not have aminocaproic acid as a specific antidote to fibrinolytic agents, so for coverage of all aspects of coagulation pathways we decided to prescribe fresh frozen plasma (FFP) and platelets, in conjunction with protamine sulfate and tranexamic acid.

**Figure 5:**
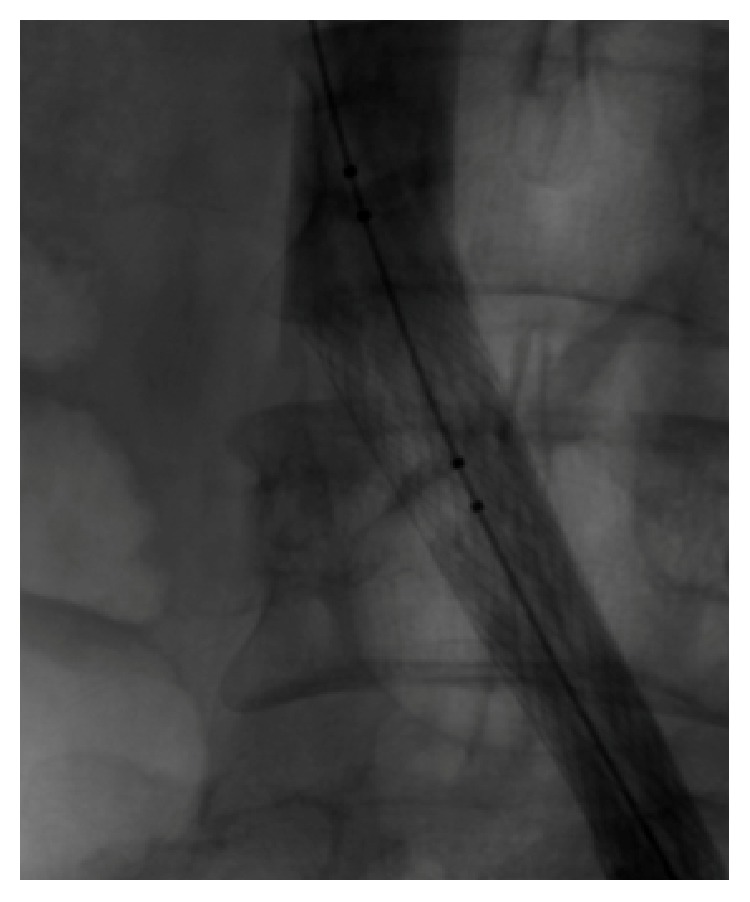
Final result of procedure.

**Figure 6:**
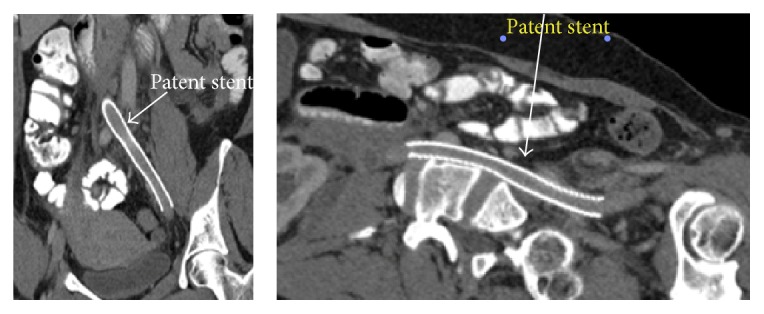
Follow-up CT scan showed patent iliac vein stent.

Fortunately, this courageous strategy in spite of fear of stent thrombosis and even our patient primary pathology itself (acute thrombosis) causes leakage to diminish significantly and in following balloon inflations to stop completely (Figure 5). After this approximately 3-hour stressful process the patient transferred in a stable and satisfactory condition to ICU. Although the next day pelvic and abdominal CT angiography showed the extent of patient bleeding and of course patent iliac vein stent (Figure 6), she passed an uneventful course in following days of admission.

## 3. Discussion

Intervention in a full anticoagulated milieu is not a safe and free of event procedure as we encountered in this case. Besides, IVC perforation is not a well-known complication, especially after thrombolytic treatment. If a serious bleeding develops in a patient who had received fibrinolytic medications, the first step is the agent cessation. The next step is to institute supportive therapy often including volume repletion and transfusion of blood factors. When possible, direct pressure should be used to control bleeding. If the patient has also been receiving heparin, protamine may be used.

Aminocaproic acid is a specific antidote to fibrinolytic agents. FFP, cryoprecipitate, or both may be used to replenish fibrin and clotting factors. Alteplase has initial half-life of 5 minutes and terminal half-life of 72 minutes but could we wait for this not short period to eliminate the drug itself while the bleeding was active and life-threatening?

## 4. Conclusion

Venous pharmacomechanical thrombectomy is an attractive technique which may result in a shorter time to vein patency, shorter length of stay, reduction in hemorrhagic risk, and overall cost savings [4, 5]. Catheter-directed thrombolysis trials long term follow-up resulted in a persistent and increased clinical benefit but not quality of life, supporting the use of additional catheter-directed thrombolysis in patients with extensive proximal DVT [6]. Although IVC perforation is not common and seems not to be as threatening as arterial counterparts perforation, this is not true in all situations. Therefore we suggest if IVC perforation occurred especially in an anticoagulated milieu, it is not wise to hesitate in hope of spontaneous stop of bleeding. Sometimes, pharmacomechanical venous intervention needs pharmacomechanical reversal of inadvertent complications.
